# Impact of post-transplantation maintenance therapy on health-related quality of life in patients with multiple myeloma: data from the Connect® MM Registry

**DOI:** 10.1007/s00277-018-3446-y

**Published:** 2018-07-29

**Authors:** Rafat Abonour, Lynne Wagner, Brian G.M. Durie, Sundar Jagannath, Mohit Narang, Howard R. Terebelo, Cristina J. Gasparetto, Kathleen Toomey, James W. Hardin, Amani Kitali, Craig J. Gibson, Shankar Srinivasan, Arlene S. Swern, Robert M. Rifkin

**Affiliations:** 1 Indiana University, Indianapolis, IN USA; 2 Hematology/Oncology, Indiana Cancer Pavilion, 535 Barnhill Drive, Suite 446, Indianapolis, IN 46202-5289 USA; 3 Wake Forest University School of Medicine, Winston-Salem, NC USA; 4 Cedars-Sinai Medical Center, Los Angeles, CA USA; 5 Mount Sinai Hospital, New York, NY USA; 6 US Oncology Research, Columbia, MD USA; 7 Providence Cancer Institute, Novi, MI USA; 8 Duke University Medical Center, Durham, NC USA; 9 Steeplechase Cancer Center, Somerville, NJ USA; 10 University of South Carolina, Columbia, SC USA; 11 Celgene Corporation, Summit, NJ USA; 12 Rocky Mountain Cancer Centers US Oncology, Denver, CO USA

**Keywords:** Registry, Multiple myeloma, Quality of life, Stem cell transplantation, Community medicine

## Abstract

Maintenance therapy after autologous stem cell transplantation (ASCT) is recommended for use in multiple myeloma (MM); however, more data are needed on its impact on health-related quality of life (HRQoL). Presented here is an analysis of HRQoL in a Connect MM registry cohort of patients who received ASCT ± maintenance therapy. The Connect MM Registry is one of the earliest and largest, active, observational, prospective US registry of patients with symptomatic newly diagnosed MM. Patients completed the Functional Assessment of Cancer Therapy-MM (FACT-MM) version 4, EuroQol-5D (EQ-5D) questionnaire, and Brief Pain Inventory (BPI) at study entry and quarterly thereafter until death or study discontinuation. Patients in three groups were analyzed: any maintenance therapy (*n* = 244), lenalidomide-only maintenance therapy (*n* = 169), and no maintenance therapy (*n* = 137); any maintenance and lenalidomide-only maintenance groups were not mutually exclusive. There were no significant differences in change from pre-ASCT baseline between any maintenance (*P* = 0.60) and lenalidomide-only maintenance (*P* = 0.72) versus no maintenance for the FACT-MM total score. There were also no significant differences in change from pre-ASCT baseline between any maintenance and lenalidomide-only maintenance versus no maintenance for EQ-5D overall index, BPI, FACT-MM Trial Outcomes Index, and myeloma subscale scores. In all three groups, FACT-MM, EQ-5D Index, and BPI scores improved after ASCT; FACT-MM and BPI scores deteriorated at disease progression. These data suggest that post-ASCT any maintenance or lenalidomide-only maintenance does not negatively impact patients’ HRQoL. Additional research is needed to verify these findings.

**Electronic supplementary material:**

The online version of this article (10.1007/s00277-018-3446-y) contains supplementary material, which is available to authorized users.

## Introduction

Multiple myeloma (MM) is a hematologic malignancy of plasma cells that has an age-adjusted incidence of 6.5 per 100,000 persons per year in the USA [1]. In 2017, an estimated 30,280 living in the USA will be diagnosed with MM, and 12,590 will die of the disease [2]. Although the rates of new MM diagnoses have been increasing at an average of 0.8% per year during the past decade, advances in the development of anti-myeloma therapies have expanded treatment options and resulted in a parallel decrease of death rates by an average of 0.8% per year [1]. However, despite the introduction of novel therapies, MM continues to be incurable and patients ultimately relapse.

Autologous stem cell transplantation (ASCT) is a standard of care for eligible patients with newly diagnosed MM (NDMM) [3–5]. Despite improvements in treatment, ASCT is not curative for most patients, with more than half of patients relapsing within 2 to 3 years after ASCT if they did not receive post-ASCT treatment [6–10]. Thus, a key treatment goal for transplant-eligible patients with NDMM is to extend post-ASCT remission. Results of analyses examining survival outcomes and tolerability associated with interferon, corticosteroid, and thalidomide maintenance therapy have been inconsistent [11–13]. Bortezomib maintenance has also been tested, with increased progression-free survival (PFS) noted [14, 15].

Findings from several randomized controlled trials with continuous lenalidomide therapy have shown significant improvements in PFS [5, 8, 10, 16] and overall survival (OS) [8], with moderate and manageable adverse event (AE) profiles. A recent meta-analysis of lenalidomide maintenance therapy post-ASCT data from three of these studies [5, 8, 10], which were not individually powered to assess OS, reported an OS benefit associated with lenalidomide maintenance therapy compared with control (no maintenance therapy) [17]. Lenalidomide maintenance therapy has been approved by the FDA and EMA and is recommended for use after ASCT in several guidelines for MM treatment. However, important questions remain, including the optimal length of maintenance treatment, patient subsets that will benefit most/least from maintenance, and the role of combination therapies for maintenance. Components of maintenance tend to emphasize survival benefit and manageable toxicities; opponents argue that the risks of long-term toxic effects outweigh the clinical benefits and advocate for the inclusion of a treatment-free interval to preserve patients’ health-related quality of life (HRQoL) [18]. Because the prolonged treatment duration of maintenance therapies requires tolerability and minimal impact on HRQoL, excessive toxicity from otherwise promising agents has previously limited the applicability of these agents in this setting [18, 19].

Patient-reported outcomes (PROs) contribute an additional valuable perspective in the ongoing maintenance discussion by quantifying the effects of long-term therapy on HRQoL directly from patients. Few HRQoL analyses of patients undergoing post-ASCT maintenance therapy have been published [20, 21], and none report real-world outcomes in community settings. The Connect MM Registry enrolled more than 3000 patients with NDMM, the vast majority (> 80%) from community settings. This registry was established as a research initiative to better understand the natural history and management of MM across community, academic, and government treatment centers. In addition to describing practice patterns, a secondary objective for the registry is to characterize the HRQoL of patients and to explore its association with treatment regimens/sequence and clinical outcomes. Data from the Connect MM registry have been used previously to establish baseline demographic and disease characteristics and to analyze the incidence of second primary malignancies among patients treated with lenalidomide [22, 23]. Presented here is an analysis of PROs from Cohort 1 of the Connect MM registry (*n* = 1493), which includes patients with NDMM who received ASCT and did or did not receive maintenance therapy during the follow-up period, to provide insights on the effects of maintenance therapy on HRQoL based on patients’ experiences.

## Patients and methods

### Study design and study population

Connect MM registry design, which has been described previously in detail (clinicaltrials.gov identifier: NCT01081028), collects longitudinal data on patients with NDMM in the USA. In order to minimize bias and better understand the representativeness of the Registry population, consecutive MM patients presenting to the sites were evaluated for potential enrollment, though participation in the registry was voluntary. All medical treatment (including medications, follow-up, and post-treatment laboratory testing) was administered at the treating physician’s discretion as per standard of care. Patients aged ≥18 years who had symptomatic NDMM within 2 months before study entry and signed informed consent were eligible for inclusion in the registry. MM diagnosis was asked to be defined per International Myeloma Working Group criteria [24]. The registry is sponsored by Celgene Corporation.

**Fig. 1:**
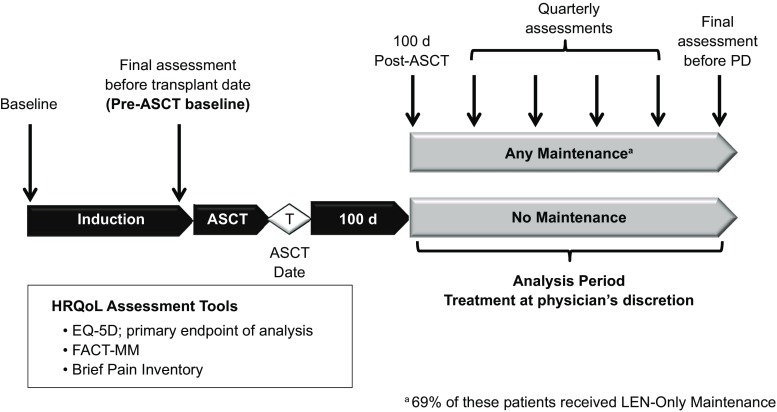
Connect MM HRQoL analysis design. Autologous stem cell transplant (ASCT), EuroQol Research Foundation EQ-5D questionnaire (EQ-5D), Functional Assessment of Cancer Therapy-Multiple Myeloma (FACT-MM); health-related quality of life (HRQoL), lenalidomide (LEN), progressive disease (PD)

The registry comprises two cohorts: Cohort 1 (*n* = 1493) includes patients enrolled from September 2009 to December 2011, and Cohort 2 (*n* = 1518) includes patients enrolled from December 2012 to April 2016. Using available site screening information, 92% of all screened patients were enrolled. Patients were followed for treatment and outcomes for as many as 8 years or until discontinuation from the study. The analysis population for the present study comprised Cohort 1 patients who completed induction therapy and first-line ASCT and had or had not received maintenance therapy post-ASCT (Fig. 1). To reduce potential sources of bias, patients who received allogeneic, tandem, or unknown types of transplant were excluded. Also, patients who received consolidation (defined as treatment received for < 60 days following transplant) before maintenance therapy were excluded from this analysis. The analysis population was categorized into three groups: (1) any type of maintenance therapy, including lenalidomide-only; (2) lenalidomide-only maintenance therapy; or (3) no maintenance therapy.

### Data collection and measures

In the Connect MM registry, PRO measures were administered to assess overall HRQoL (Functional Assessment of Cancer Therapy–General; FACT-G), myeloma-specific concerns (FACT-Multiple Myeloma subscale; FACT-MM), pain severity (Brief Pain Inventory; BPI), and health utilities (EuroQol Research Foundation EQ-5D questionnaire) [25–28]. PROs were completed in the clinic at study enrollment (study baseline) and approximately quarterly (based on frequency of clinic visits) thereafter until the end of the study’s follow-up period, early study discontinuation, or death. In efforts to minimize the variability of HRQoL scores among patients who are treated at their physicians’ discretion (proving more difficult to collect HRQoL assessments closer to the initiation of maintenance therapy), HRQoL assessments analyzed were those collected at study baseline, after induction therapy but prior to ASCT (analytical baseline or t0), and quarterly from 100 days post-ASCT until the end of maintenance therapy or until progressive disease, discontinuation, or death (analytic period).

The FACT-MM questionnaire consists of the four core FACT HRQoL subscales measuring physical, functional, social, and emotional well-being (FACT-G; 27 items) and an additional subscale (MM subscale) measuring MM-specific concerns (14 items). Following standard FACT instructions, items were rated using a 0- to 4-point scale based on the past 7 days, with higher scores indicating better HRQoL and fewer MM-related symptoms. Three scores were calculated for analysis: the FACT-MM total score (all domains; scale, 0–164), the trial outcome index (TOI; physical, functional, and MM-specific domains; scale, 0–112), and an MM subscale score (scale, 0–56). The EQ-5D is a general, nondisease measure of health utilities and assesses five dimensions: mobility, self-care, usual activities, pain/discomfort, and anxiety/depression. Summary assessments for EQ-5D use the visual analog scale and an index score (scale, − 0.109 to 1), with higher scores indicating better health states. The BPI short form assesses the existence and intensity of pain on a scale of 0 to 10 (none to worst) and categorizes pain as mild (1–4), moderate (5–6), and severe (7–10).

The completion rates for each HRQoL instrument and time point were calculated as the number of patients who completed the instrument divided by the number of patients who had not discontinued or died by that time point. The analysis was conducted using a mixed model which is robust under missing at random (MAR) assumptions.

### Statistical analysis

SAS Proc Mixed with a random effects unstructured covariance matrix to estimate mixed regression models was used to test the null hypothesis of no HRQoL difference between patients receiving any maintenance versus no maintenance therapy, and patients receiving lenalidomide-only maintenance versus no maintenance therapy. A quadratic growth model was applied with time as a continuous variable (given that ASCT can occur at any fractional quarterly period post-enrollment and having started at 100 days post-ASCT) adjusted for potential confounders including: study baseline renal impairment and presence of del(17p); analytic baseline history of monoclonal gammopathy of undetermined significance and peripheral neuropathy; day-100 post-ASCT albumin; and first-regimen first-course treatment of novel therapy (immunomodulatory agent or protease inhibitor), triplet therapy, and lenalidomide. The complete list of variables included in the analysis is listed in Table 5 (Online Resource 1). A post-hoc power assessment was conducted to determine whether the study was powered to find differences in PROs. For any maintenance (*n* = 244) or lenalidomide-only maintenance (*n* = 169) vs no maintenance (*n* = 137): 99% power to detect minimal clinically important differences in HRQoL scales with two-sided *P* value of 0.05.

#### Data availability

The datasets generated during and/or analyzed during the current study are available from the corresponding author on reasonable request.

## Results

### Patient characteristics

**Fig. 2:**
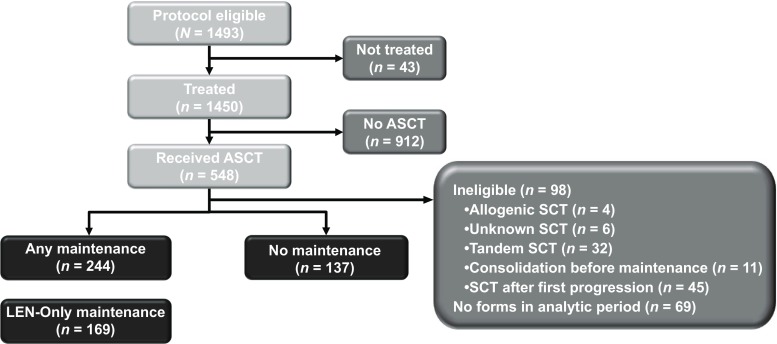
Patient disposition. Autologous stem cell transplant (ASCT), lenalidomide (LEN). ^a^Patients were excluded if they received allogenic or unknown stem cell transplant (SCT), tandem SCT, or consolidation before maintenance in course 1 or if they received SCT after first progression

Between September 2009 and December 2011, 1493 patients had enrolled in Cohort 1 of the Connect MM registry from community (81%), academic (18%), or government (1%) centers. The mean time from initial diagnosis to enrollment in this registry was 25 days. Of the 1493 patients enrolled, 548 patients received ASCT; of these, 244 met the analysis criteria for any maintenance, 169 for lenalidomide-only maintenance, and 137 for no maintenance (Table 1; Fig. 2). At study entry, the median age was 60 years (range, 24–78 years), 61% were men, and 85% were white. Most patients had an Eastern Cooperative Oncology Group performance status of 0 or 1 (64%) and were International Staging System stage I or II (57%). A higher percentage of patients in the groups receiving maintenance therapy had received triplet therapy as induction therapy (63 and 65% for any maintenance or lenalidomide-only maintenance therapies, respectively), compared with the group receiving no maintenance therapy (50%). Table 6 (Online Resource 1) provides the breakdown of types of maintenance therapies administered in the group receiving any maintenance therapy.

**Table 1:** Patient characteristics

Characteristic	Any maintenance therapy (*n* = 244)	Lenalidomide-only maintenance therapy (*n* = 169)	No maintenance therapy (*n* = 137)
Age (years)			
Median (range)	60 (24–78)	60 (24–74)	60 (27–75)
< 65	166 (68.0)	120 (71.0)	96 (70.1)
65 to <75	75 (30.7)	49 (29.0)	40 (29.2)
Men	154 (63.1)	106 (62.7)	77 (56.2)
Race			
White	209 (85.7)	144 (85.2)	114 (83.2)
Black	28 (11.5)	20 (11.8)	16 (11.7)
ECOG PS			
0–1	149 (61.0)	111 (65.7)	95 (69.3)
2–3	20 (8.2)	9 (5.4)	7 (5.1)
Not specified	75 (30.7)	49 (29.0)	35 (25.5)
ISS stage^a^			
I	72 (29.5)	53 (31.4)	42 (30.7)
II	68 (27.9)	50 (29.6)	31 (22.6)
III	54 (22.1)	32 (18.9)	35 (25.5)
Not specified	50 (20.5)	34 (20.1)	29 (21.2)
Type of induction therapy			
Lenalidomide-containing	143 (58.6)	108 (63.9)	72 (52.6)
Bortezomib-containing	209 (85.7)	141 (83.4)	108 (78.8)
Alkylator-containing	44 (18.0)	27 (16.0)	15 (10.9)
Novel agents	243 (99.6)	168 (99.4)	133 (97.1)
Triplet	153 (62.7)	110 (65.1)	69 (50.4)
IMWG risk			
Low	28 (11.5)	25 (14.8)	16 (11.7)
Standard	93 (38.1)	64 (37.9)	57 (41.6)
High	50 (20.5)	33 (19.5)	20 (14.6)
Missing/not specified	73 (29.9)	47 (27.8)	44 (32.1)
Creatinine category			
> 2.0 mg/dL	27 (11.1)	17 (10.1)	23 (16.8)
≤ 2.0 mg/dL	217 (88.9)	152 (89.9)	114 (83.2)
Albumin^b^			
< 3.5 g/dL	26 (10.7)	16 (9.5)	24 (17.5)
≥ 3.5 g/dL	173 (70.9)	123 (72.8)	89 (65.0)
Abnormal platelet count (≤ 150 × 10^9^/L)	63 (25.8)	40 (23.7)	40 (29.2)
Neutropenia (ANC ≤ 1.5 × 10^9^/L)	21 (8.6)	11 (6.5)	13 (9.5)
Anemia (Hb < 10 g/dL)	23 (9.4)	12 (7.1)	12 (8.8)

Abbreviations: *ANC* absolute neutrophil count, *ASCT* autologous stem cell transplant, *ECOG* Eastern Cooperative Oncology Group, *Hb* hemoglobin, *IMWG* International Myeloma Working Group, *ISS* International Staging System, *PS* performance status

Values shown are *n* (%) unless otherwise indicated

^a^As defined in: Greipp PR, et al. [31]

^b^Data provided for albumin are for 100 days post-ASCT

### HRQoL questionnaires completion rates and baseline scores

Median follow-up time was 39.3 months (range, 0.0–87.1 months) at the time of data cutoff (July 2016). The median duration of maintenance treatment was 23.5 months (range, 0.6–69.6 months) in the group receiving any maintenance therapy and 24.9 months (range, 0.6–69.6 months) in the group receiving lenalidomide-only maintenance therapy (Table 2). During the analysis period, the completion rate for the FACT-MM was higher in the non-maintenance group (odds ratio 1.4; Table 2); results were similar for the other tools. Increased completion rates may be due to closer follow-up and thus more frequent clinic visits in patients not receiving maintenance. However, among high completers (> 75% of forms) and low completers (< 25% of expected forms), there were no differences in baseline characteristics (e.g. age, race, sex, disease stage, performance status, renal insufficiency); slight differences in completion were seen between academic and community settings (data not shown). Mean analysis baseline scores for the EQ-5D, FACT-MM (total, TOI, and MM subscale), and BPI were similar across groups (Table 2).

**Table 2:** HRQoL completion and baseline scores

	Any maintenance therapy (*n* = 244)	Lenalidomide-only maintenance therapy (*n* = 169)	No maintenance therapy (*n* = 137)
Median (range) duration of maintenance (months)	23.5 (0.6–69.6)	24.9 (0.6–69.6)	NA
EQ-5D completion rate, *n*/*N* (%)^a^			
Study baseline	241/244 (98.8)	167/169 (98.8)	136/137 (99.3)
Quarter 1	133/243 (54.3)	94/168 (56.0)	107/137 (78.1)
Quarter 2	156/223 (70.0)	110/154 (71.4)	99/123 (80.5)
Quarter 8^b^	75/116 (64.7)	56/83 (67.5)	48/63 (76.2)
Mean (SD) analysis baseline HRQoL score			
FACT-MM total score	118.0 (23.5)	118.9 (23.3)	118.5 (24.1)
FACT-MM trial outcomes index	75.8 (18.5)	76.4 (18.6)	76.0 (19.3)
FACT-MM MM subscale	38.2 (9.7)	38.3 (9.9)	38.9 (9.4)
EQ-5D overall index	0.79 (0.14)	0.79 (0.14)	0.79 (0.14)
BPI	4.0 (2.4)	4.0 (2.4)	3.9 (2.5)

*ASCT* autologous stem cell transplant, *BPI* Brief Pain Inventory, *EQ-5D* EuroQol Research Foundation EQ-5D questionnaire, *FACT-MM* Functional Assessment of Cancer Therapy-Multiple Myeloma, *HRQoL* health-related quality of life, *NA* not applicable, *SD* standard deviation

^a^Completion rates for all instruments were similar because measures were administered at the same times

^b^Data through quarter 8 was selected to correspond with the ~ 24-month median duration of maintenance therapy

### HRQoL analyses

**Fig. 3:**
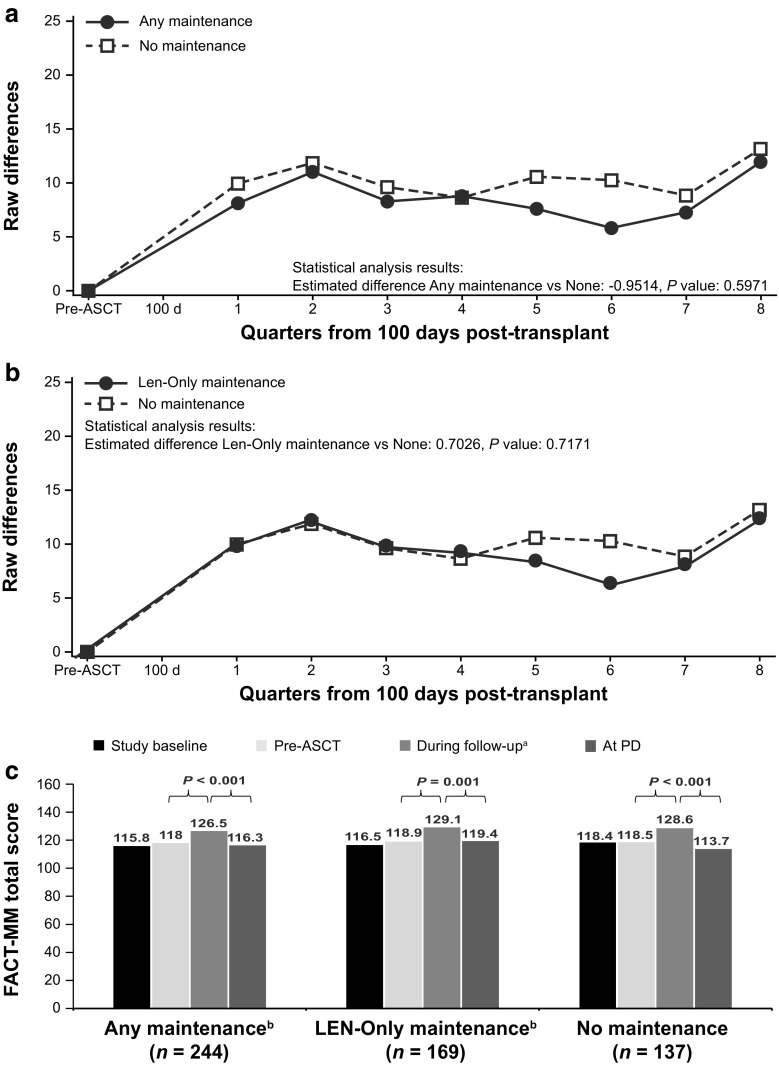
FACT-MM total score change from pre-ASCT baseline (adjusted) values. FACT-MM total score scale is 0 to 164. **a** Any maintenance therapy (solid line) versus no maintenance therapy (dashed line). **b** Lenalidomide-only maintenance therapy (solid line) versus no maintenance therapy (dashed line). **c** HRQoL at baseline, pre-ASCT, during follow-up, and at PD. Autologous stem cell transplant (ASCT), Functional Assessment of Cancer Therapy-Multiple Myeloma (FACT-MM), health-related quality of life (HRQoL), lenalidomide (LEN), least-squares (LS), progressive disease (PD).^.a^LS mean during the analysis period, ^b^66 patients had PD

**Fig. 4:**
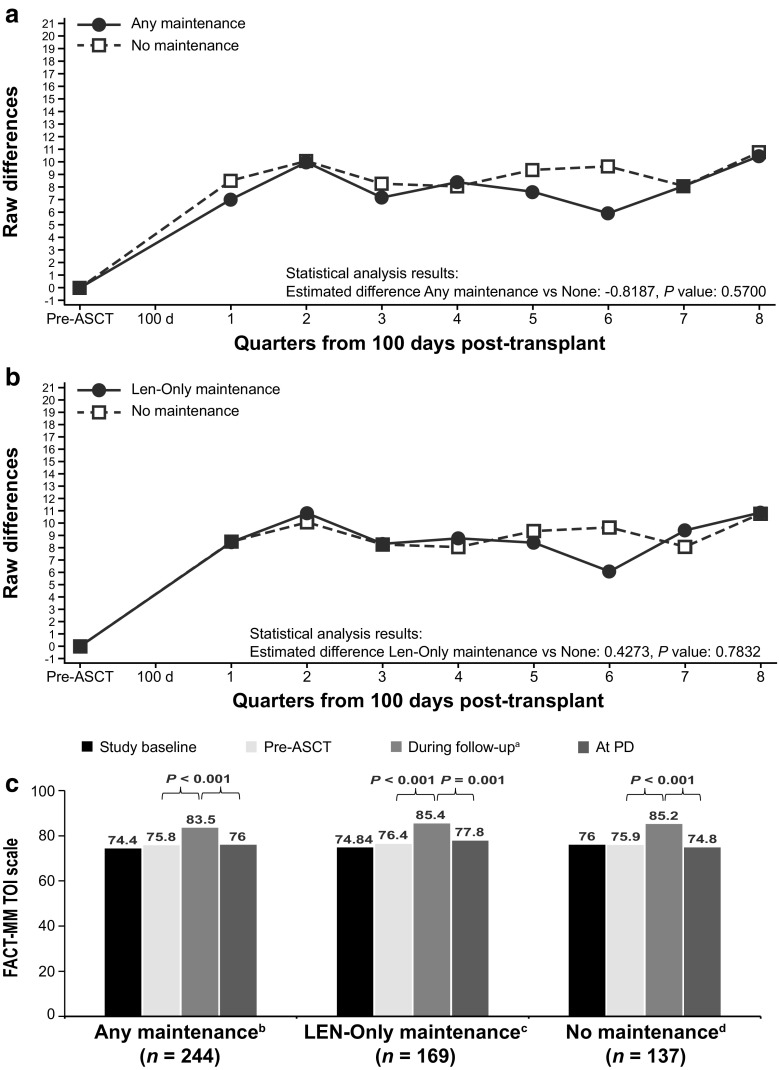
FACT-MM TOI change from pre-ASCT baseline values (adjusted). FACT-MM TOI scale is 0 to 112. **a** Any maintenance therapy (solid line) versus no maintenance therapy (dashed line). **b** Lenalidomide-only maintenance therapy (solid line) versus no maintenance therapy (dashed line). **c** HRQoL at baseline, pre-ASCT, during follow-up, and at PD *P* values are paired *t* test comparisons between periods within treatment group. The TOI is the sum of the physical well-being, functional well-being, and “additional concerns” subscores. Autologous stem cell transplant (ASCT), Functional Assessment of Cancer Therapy-Multiple Myeloma (FACT-MM), health-related quality of life (HRQoL), lenalidomide (LEN), least-squares (LS), progressive disease (PD), trial outcome index (TOI). ^a^LS mean during the analysis period, ^b^66 patients had PD, ^c^45 patients had PD, ^d^44 patients had PD

**Fig. 5:**
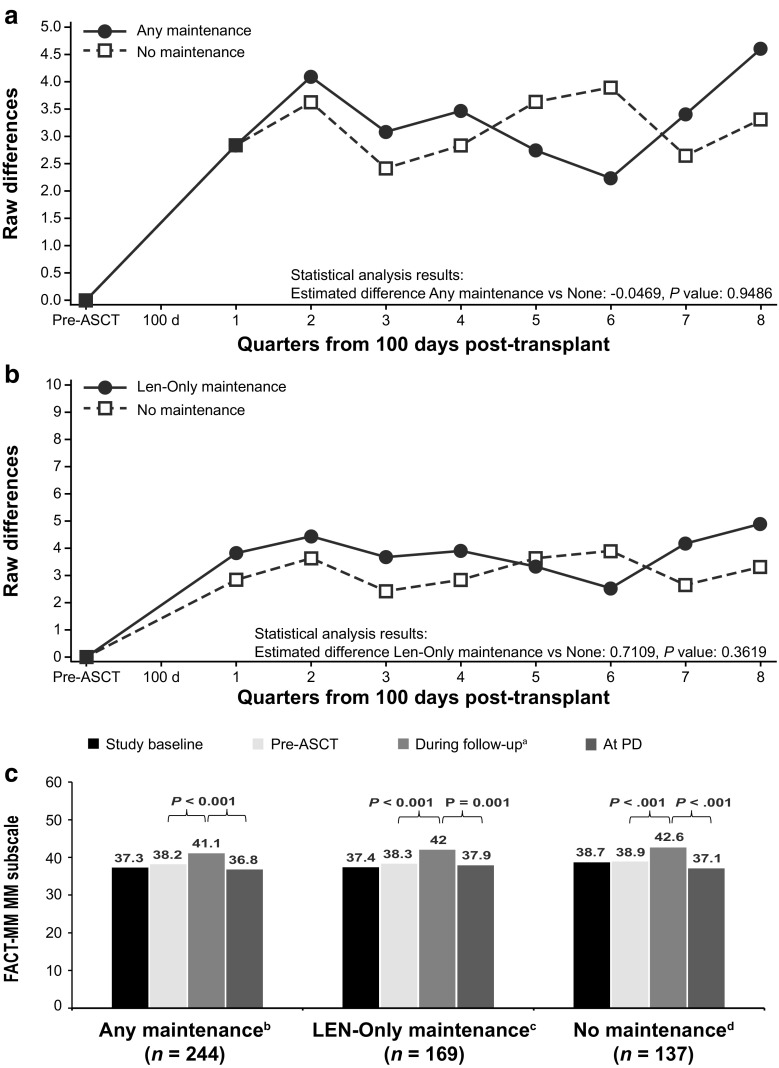
FACT-MM MM subscale change from pre-ASCT baseline values (adjusted). FACT-MM MM subscale is 0 to 56. **a** Any maintenance therapy (solid line) versus no maintenance therapy (dashed line). **b** lenalidomide-only maintenance therapy (solid line) versus no maintenance therapy (dashed line). **c** HRQoL at baseline, pre-ASCT, during follow-up, and at PD. *P* values are paired *t.*test comparisons between periods within treatment group. Autologous stem cell transplant (ASCT), Functional Assessment of Cancer Therapy-Multiple Myeloma (FACT-MM), health-related quality of life (HRQoL), lenalidomide (LEN), least-squares (LS), progressive disease (PD). ^a^LS mean during the analysis period. ^b^66 patients had PD. ^c^45 patients had PD. ^d^44 patients had PD

No statistically significant differences in change from pre-ASCT baseline values were observed between the groups receiving any maintenance therapy and lenalidomide-only maintenance therapy versus the group receiving no maintenance therapy for the FACT-MM total score (Fig. 3a, b), TOI score (Fig. 4a, b), and myeloma subscale score (Fig. 5a, b; Table 7 [Online Resource 1]). Across the treatment groups, FACT-MM total score, TOI score, and MM subscale score increased significantly from pre-ASCT to the follow-up period (*P* < 0.001 for all groups) and decreased significantly at progression (*P <* 0.01, all groups; Figs. 3c, 4c, and 5c).

**Fig. 6:**
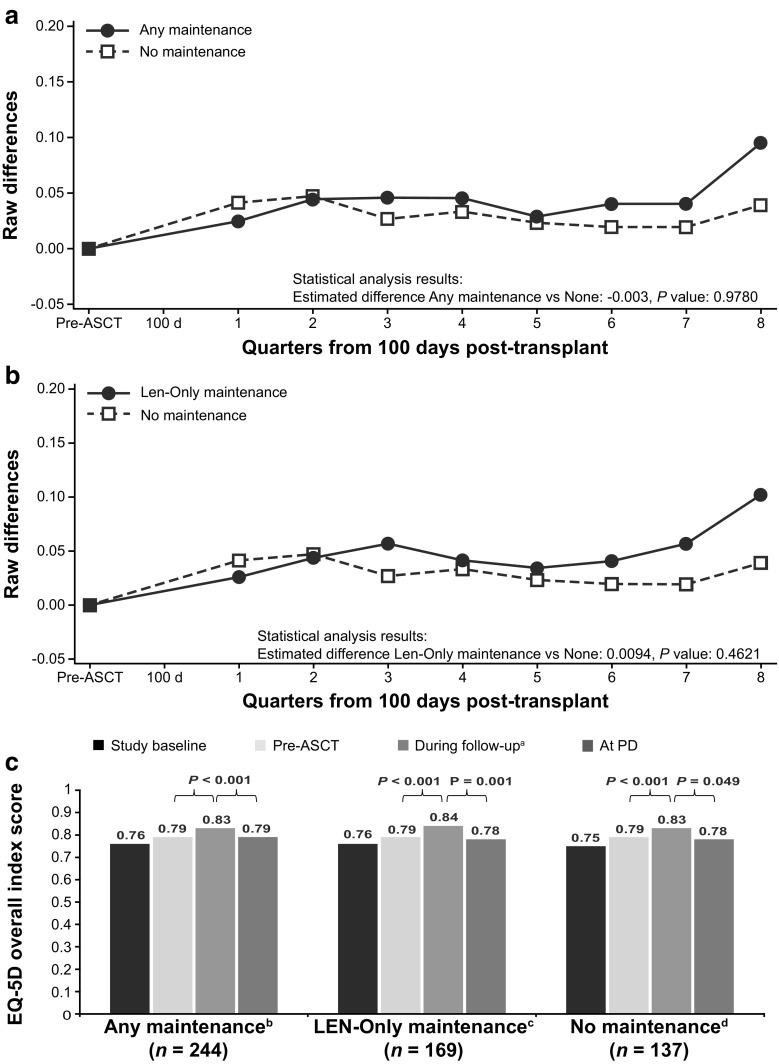
EQ-5D overall index score change from pre-ASCT baseline values (adjusted). EQ-5D score scale is − 0.109 to 1. **a** Any maintenance therapy versus no maintenance therapy. **b** Lenalidomide-only maintenance therapy versus no maintenance therapy. **c** HRQoL at baseline, pre-ASCT, during follow-up, and at PD. *P* values are paired *t* test comparisons between periods within treatment group. Autologous stem cell transplant (ASCT), EuroQol Research Foundation questionnaire (EQ-5D), lenalidomide (LEN), least-squares (LS), progressive disease (PD). ^a^LS mean during the analysis period, ^b^44 patients in the LEN-only maintenance group had PD

There were no statistically significant differences in change in EQ-5D overall index scores over time between the group receiving no maintenance therapy and the groups receiving any maintenance (*P* = 0.98) or lenalidomide-only maintenance therapy (*P* = 0.46; Fig. 6a, b; Table 7 [Online Resource 1]). Across groups, EQ-5D overall index score increased significantly from pre-ASCT to the follow-up period (*P* < 0.001 for all groups) and decreased at progression (Fig. 6c). The decrease at progression reached statistical significance in the groups receiving lenalidomide-only maintenance therapy (*P* = 0.001; Fig. 6c) and no maintenance therapy (*P* = 0.049).

Change in BPI scores from baseline also did not differ significantly between the groups for those receiving any maintenance therapy versus no maintenance therapy (*P* = 0.81) and those receiving lenalidomide-only maintenance therapy versus no maintenance therapy (*P* = 0.42; Fig. 7a and b; Table 7 [Online Resource 1]). BPI decreased significantly from pre-ASCT (analytic baseline) to follow-up for the groups receiving any maintenance therapy and lenalidomide-only maintenance therapy and significantly increased at progression for all groups (*P* < 0.001; Fig. 7c [Online Resource 1]).

## Discussion

The findings from this analysis of PRO data from the Connect MM patient registry showed no deterioration in HRQoL, despite continued active therapy in the maintenance therapy groups. Patient-reported HRQoL using the FACT-MM, EQ-5D Index, and BPI improved after transplant in all three groups and numerically deteriorated on progression. Deterioration of HRQoL scores was significant when assessed with the FACT-MM total score and specific subscales (TOI and MM-Scale). Interestingly, HRQoL was measured before patients being informed of disease progression; thus, HRQoL deterioration was observed independently. A trend towards greater improvement in HRQoL was observed in the groups receiving any maintenance therapy and lenalidomide-only maintenance therapy, as compared with the group receiving no maintenance therapy. Results were consistent across all HRQoL measures.

Given that prolonged therapy is associated with improved prognosis, understanding therapy-related decrements to HRQoL has become increasingly important. Key considerations when deciding whether maintenance therapy should be recommended could include an individual’s risk factors and depth of response, weighed by the potential for toxicities that accompany continued treatment and potential HRQoL impairments.

Results of phase 3 randomized trials have shown consistently that post-ASCT lenalidomide maintenance therapy significantly extends the duration of remission as compared with no maintenance therapy [5, 8, 10, 29]. Recently, a meta-analysis of three phase 3 trials showed a significant OS benefit for lenalidomide maintenance therapy versus no maintenance therapy [17]. With significant improvements in PFS, results of the Myeloma XI study (*N* = 1550) support use of maintenance lenalidomide as standard of care regardless of patient age [22]. Although rates of hematologic AEs and discontinuation because of AEs reported have been higher for patients receiving lenalidomide maintenance therapy compared with placebo, the general toxicity profile is manageable [8, 10]. Second primary malignancies were higher for patients receiving lenalidomide maintenance therapy; however, when compared with the meaningful survival improvement, the benefit–risk profile remained positive [17]. However, PROs were not collected in these studies; therefore, HRQoL outcomes were not adequately assessed.

Connect MM is the first and largest prospective registry of patients with NDMM in the USA. The collection of longitudinal data on clinical practice and treatment outcomes in both clinical and nonclinical trial settings among patients predominantly treated in community-based practices provides a rich understanding of real-world clinical practices. Because of the large volume of data, findings from this study reflect real-world clinical practice; therefore, the generalizability of findings and potential to inform patient care represent major strengths. Registry study analyses can be confounded by limitations in data entry or data reporting and by the observational, nonrandomized nature of the study. Since these patients were treated at their physicians’ discretion, using HRQoL assessments collected immediately prior to the initiation of maintenance therapy could increase the variability of HRQoL scores among patients; therefore, analytical baseline for HRQoL assessments was immediately prior to ASCT. Post-baseline assessments began at 100 days post-ASCT and were conducted quarterly. However, our post-baseline completion rates for HRQoL instruments (ranging from 56 to 82% for EQ-5D through Quarter 8) were similar to those reported in a phase 3 clinical trial (MM-015) of lenalidomide maintenance therapy versus no maintenance therapy after lenalidomide-melphalan-prednisone treatment in older patients (aged ≥ 65 years) with newly diagnosed MM [30], as well as in a phase 3 trial (MM-020) of lenalidomide-dexamethasone versus melphalan-prednisone-thalidomide in newly diagnosed MM (EQ-5D 65–92% through month 18; data on file). Another limitation of this study is that the impact of AEs on PROs was not assessed. Finally, there is a drop-off in patients with clinical and HRQoL data during the follow-up period (as is expected for a registry study); however, patient demographics and disease characteristics were similar at baseline between those completing and not completing HRQoL assessments.

In conclusion, these results show that post-ASCT maintenance therapy, including lenalidomide-only maintenance therapy, did not negatively affect HRQoL in patients in the Connect MM Registry. Patients had generally similar scores on measures assessing overall HRQoL, myeloma-specific concerns, pain severity, and health utilities, regardless of maintenance or no maintenance therapy after ASCT. These findings suggest that continued, active, post-ASCT maintenance therapy does not decrease HRQoL while improving clinical outcomes, thus supporting a favorable benefit–risk profile. Confirmation of these outcomes with a longer follow-up and in the Connect MM registry Cohort 2 is warranted.

## Electronic supplementary material

ESM 1 (DOCX 270 kb)
