# Supplementary material for: Impact of post-transplantation maintenance therapy on health-related quality of life in patients with multiple myeloma: data from the Connect® MM Registry
Source: Ann Hematol. 2018 Jul 29;97(12):2425–36. doi: 10.1007/s00277-018-3446-y (PMC6208675; doi:10.1007/s00277-018-3446-y)
Supplement: Supplementary file 1 — (DOCX 270 kb) [file 277_2018_3446_MOESM1_ESM.docx]

# ONLINE RESOURCE 1. Figure 7 and Tables 5, 6 and 7

**Fig. 7** BPI change from pre-ASCT baseline values (adjusted)**.** BPI scale is 1 to 11. **a** Any maintenance (solid line) versus no maintenance (dashed line); **b** lenalidomide-only maintenance (solid line) versus no maintenance (dashed line); **c** comparison of BPI over time. ^a^LS mean during the analysis period; ^b^66 patients had PD. Autologous stem cell transplant (ASCT); Brief Pain Inventory (BPI), lenalidomide (LEN), least-squares (LS), progressive disease (PD)

**
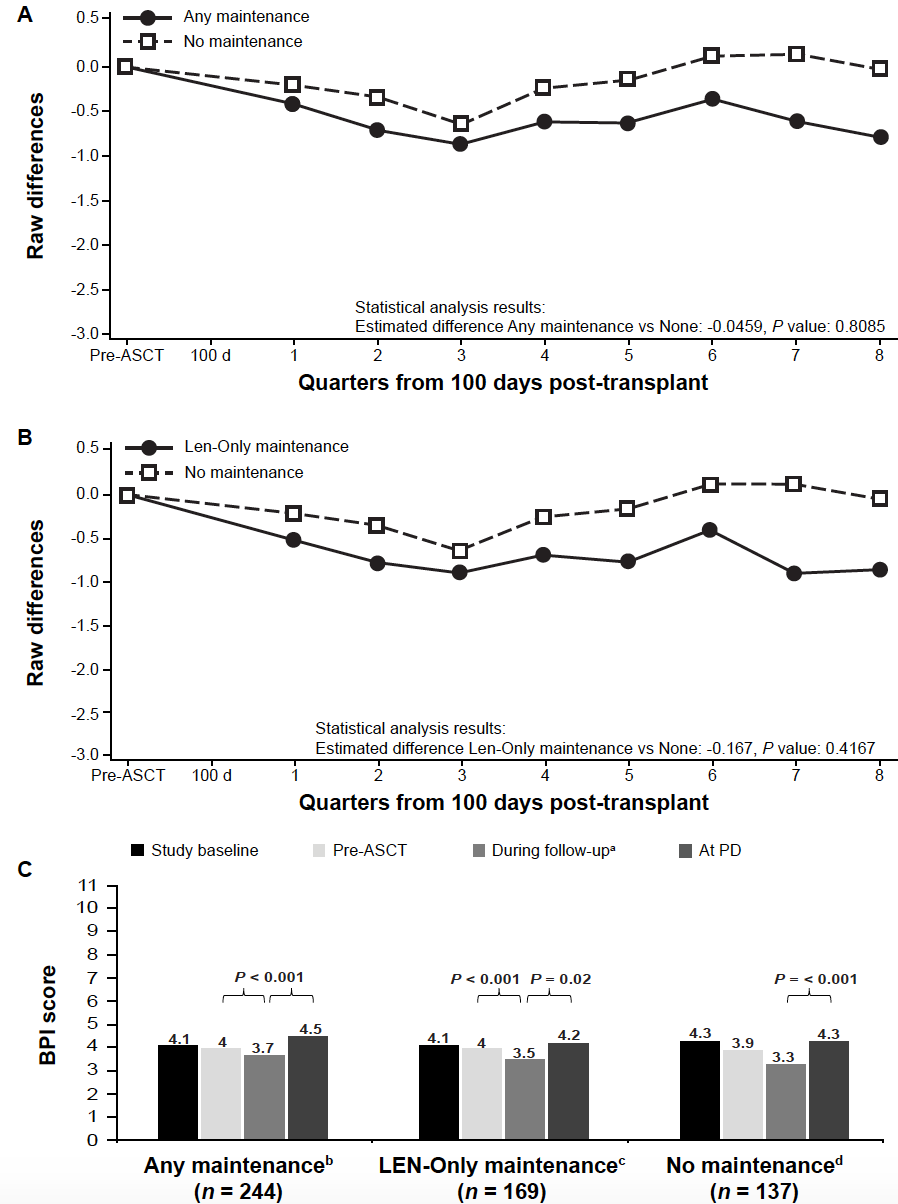
**

**Table 5.** Variables from quadratic growth model analysis of HRQoL differences across treatment groups

| **Variable** | ***P*-value** | **Variable** | ***P*-value** |
| --- | --- | --- | --- |
| Calcium | 0.1144 | Gender | 0.248 |
| CRAB prior first-line criteria | 0.3067 | Triplet agent treatment | 0.0096 |
| Creatinine category | 0.0854 | Transplant | 0.8528 |
| History of MGUS | 0.0191 | Lenalidomide treatment | 0.0454 |
| History of smoldering MM | 0.5698 | Bortezomib treatment | 0.3051 |
| History of asymptomatic MM | 0.8538 | Age groups | 0.755 |
| ECOG performance status | 0.8554 | Albumin | 0.7844 |
| del(17p), from FISH | 0.0859 | ß2M | 0.2026 |
| Hyperdiploidy | 0.5785 | Body mass index | 0.5882 |
| t(4;14) | 1 | MM bone involvement | 0.7773 |
| Hemoglobin category | 0.8458 | Neutropenia | 0.0369 |
| IMWG risk | 0.4372 | IgG immunoglobulin | 0.9155 |
| ISS stage | 0.1326 | Lactic acid hydrogenase | 0.5003 |
| History of amyloidosis | 0.8816 | Mobility from EQ-5D | 0.3339 |
| History of diabetes | 0.9619 | Surgery for MM | 0.7637 |
| Evidence of extramedullary plasmacytoma | 0.3054 | Serum free light chain level abnormality | 0.9261 |
| History of hypertension requiring treatment | 0.9861 | Self-care from EQ-5D | 0.5739 |
| Family history of MM | 0.9681 | ECOG 100 Days post-SCT | 0.9305 |
| Family history of other cancers | 0.8842 | Albumin category 100 days post-SCT | 0.0381 |
| History of peripheral neuropathy | 0.0574 | ANC category 100 days post-SCT | 0.3103 |
| History of solitary plasmacytoma | 0.519 | ß2M category 100 days post-SCT | 0.6235 |
| Past history of VTE | 0.9768 | Creatinine category 100 days post-SCT | 0.3117 |
| Non-secretory MM | 0.0972 | Hemoglobin category 100 days post-SCT | 0.5442 |
| Novel agent | 0.0095 | Platelet category 100 days post-SCT | 0.2297 |
| Platelet count | 0.4055 | del(17p) group 100 days post-SCT | 0.2604 |
| Radiation therapy for MM | 0.7403 |  |  |

ANC, absolute neutrophil count; ß2M, ß2 microglobulin; CRAB, increased calcium level, renal dysfunction, anemia, and destructive bone lesions; ECOG, Eastern Cooperative Oncology Group; EQ-5D, EuroQol-5D; FISH, fluorescence in situ hybridization; IgG, Immunoglobulin G; IMWG, International Myeloma Working Group; ISS, International Staging System; MGUS, monoclonal gammopathy of undetermined significance; MM, multiple myeloma; VTE, venous thromboembolism.

**Table 6** Maintenance therapies received in the any maintenance therapy group

| **Maintenance therapy** | **Any maintenance (*n* = 244)** |
| --- | --- |
| Lenalidomide | 169 (69.3) |
| Lenalidomide + dexamethasone | 23 (9.4) |
| Bortezomib | 17 (6.9) |
| Lenalidomide + bortezomib + dexamethasone | 12 (4.9) |
| Bortezomib + dexamethasone | 7 (2.9) |
| Lenalidomide + bortezomib | 5 (2) |
| Thalidomide | 3 (1.2) |
| Other | 8 (3.3) |
| Total | 244 (100.0) |

Values shown are *n* (%).

**Table 7** Summary of HRQoL comparisons between the 3 maintenance therapy groups: Any, Len-only, and no maintenance

| **HRQoL measure** | **Comparison vs. no maintenance** | **Estimated differences on change from baseline  LS mean (95% CI)** | ***P* value** |
| --- | --- | --- | --- |
| FACT-MM total score  (scale, 0-164) | Any | −0.951(−4.488 to 2.585) | 0.60 |
|  | LEN-only | 0.703(−3.107 to 4.512) | 0.72 |
| EQ-5D overall index  (scale, −0.109 to 1) | Any | −0.0003(−0.024 to 0.023) | 0.98 |
|  | LEN-only | 0.009 (−0.016 to 0.035) | 0.46 |
| FACT-MM TOI  (scale, 0-112) | Any | −0.81 (−3.650 to 2.0127) | 0.57 |
|  | LEN-only | 0.427(−2.624 to 3.479) | 0.78 |
| FACT-MM MM subscale  (scale, 0-56) | Any | −0.047(−1.477 to 1.383) | 0.95 |
|  | LEN-only | 0.711(−0.820 to 2.242) | 0.36 |
| BPI  (scale, 1-11) | Any | −0.046(−0.418 to 0.326) | 0.81 |
|  | LEN-only | −0.167(−0.570 to 0.237) | 0.42 |
| *BPI* brief pain inventory, *CI* confidence interval, *EQ-5D* EuroQol-5D, *FACT-MM* Functional Assessment of Cancer Therapy-Multiple Myeloma, *HRQoL* health-related quality of life, *LEN* lenalidomide; *LS* least squares, *TOI* trial outcome index. | | | |
